# Artificial neural network classifier predicts neuroblastoma patients’ outcome

**DOI:** 10.1186/s12859-016-1194-3

**Published:** 2016-11-08

**Authors:** Davide Cangelosi, Simone Pelassa, Martina Morini, Massimo Conte, Maria Carla Bosco, Alessandra Eva, Angela Rita Sementa, Luigi Varesio

**Affiliations:** 1 Laboratory of Molecular Biology, Gaslini Institute, Largo G. Gaslini 5, 16147 Genoa, Italy; 2 Department of Hematology-Oncology, Gaslini Institute, Largo G. Gaslini 5, 16147 Genoa, Italy; 3 Department of Pathology, Gaslini Institute, Largo G. Gaslini 5, 16147 Genoa, Italy

**Keywords:** Neuroblastoma, Hypoxia, Outcome prediction, Gene set enrichment analysis, Gene signature

## Abstract

**Background:**

More than fifty percent of neuroblastoma (NB) patients with adverse prognosis do not benefit from treatment making the identification of new potential targets mandatory. Hypoxia is a condition of low oxygen tension, occurring in poorly vascularized tissues, which activates specific genes and contributes to the acquisition of the tumor aggressive phenotype. We defined a gene expression signature (NB-hypo), which measures the hypoxic status of the neuroblastoma tumor. We aimed at developing a classifier predicting neuroblastoma patients’ outcome based on the assessment of the adverse effects of tumor hypoxia on the progression of the disease.

**Methods:**

Multi-layer perceptron (MLP) was trained on the expression values of the 62 probe sets constituting NB-hypo signature to develop a predictive model for neuroblastoma patients’ outcome. We utilized the expression data of 100 tumors in a leave-one-out analysis to select and construct the classifier and the expression data of the remaining 82 tumors to test the classifier performance in an external dataset. We utilized the Gene set enrichment analysis (GSEA) to evaluate the enrichment of hypoxia related gene sets in patients predicted with “Poor” or “Good” outcome.

**Results:**

We utilized the expression of the 62 probe sets of the NB-Hypo signature in 182 neuroblastoma tumors to develop a MLP classifier predicting patients’ outcome (NB-hypo classifier). We trained and validated the classifier in a leave-one-out cross-validation analysis on 100 tumor gene expression profiles. We externally tested the resulting NB-hypo classifier on an independent 82 tumors’ set. The NB-hypo classifier predicted the patients’ outcome with the remarkable accuracy of 87 %. NB-hypo classifier prediction resulted in 2 % classification error when applied to clinically defined low-intermediate risk neuroblastoma patients. The prediction was 100 % accurate in assessing the death of five low/intermediated risk patients. GSEA of tumor gene expression profile demonstrated the hypoxic status of the tumor in patients with poor prognosis.

**Conclusions:**

We developed a robust classifier predicting neuroblastoma patients’ outcome with a very low error rate and we provided independent evidence that the poor outcome patients had hypoxic tumors, supporting the potential of using hypoxia as target for neuroblastoma treatment.

**Electronic supplementary material:**

The online version of this article (doi:10.1186/s12859-016-1194-3) contains supplementary material, which is available to authorized users.

## Background

Neuroblastoma is the most common pediatric solid tumor of the sympathetic nervous system deriving from ganglionic lineage precursors [1]. It is diagnosed during infancy and shows notable heterogeneity with regard to both histology and clinical behavior [2, 3], ranging from rapid progression associated with metastatic spread and poor clinical outcome to spontaneous, or therapy-induced, regression into benign ganglioneuroma [4]. Age at diagnosis, International Neuroblastoma Staging System (INSS stage), histology, grade of differentiation, chromosomal aberrations, and amplification of the Myelocytomatosis viral related oncogene Neuroblastoma derived (MYCN) are clinical and molecular risk factors [2, 5, 6] commonly combined to classify patients into high, intermediate and low risk subgroups on which current therapeutic strategy is based [7, 8]. Although the survival of children with neuroblastoma improved over the last 25 years [9], more than fifty percent of patients with adverse prognosis do not get benefit from treatment making the exploration of new therapeutic approaches and the identification of new potential targets mandatory [10]. Patients with localized tumors have a more favorable outcome although the survival of stage 3 patients does not exceed 67 % [9]. The progression of localized tumors is closely associated to their growth rather than to their metastatic spread and understanding the molecular program at the time of diagnosis may be the key for improving the stratification and deciding the correct therapy.

The availability of neuroblastoma genomic profiles improved our prognostic ability. Several groups have developed gene expression-based approaches to stratify neuroblastoma patients [11–28] and described prognostic gene signatures. We studied outcome prediction in neuroblastoma patients utilizing a biology-driven approach, in which the gene expression profile under investigation is associated to “a priori” knowledge of a biological process that has a major impact on tumor growth [29]. Specifically, we studied the response of neuroblastoma to hypoxia and used this information to derive a novel prognostic signature [12, 29].

Hypoxia, a condition of low oxygen tension occurring in poorly vascularized areas, has profound effects on tumor cell growth, genotype selection, susceptibility to apoptosis and resistance to radio- and chemotherapy, tumor angiogenesis, epithelial to mesenchymal transition and propagation of cancer stem cells [30–33]. Hypoxia activates specific genes encoding angiogenic, metabolic and metastatic factors [31, 34, 35] and contributes to the acquisition of the tumor aggressive phenotype [31, 36–38]. We derived a 62-probe set neuroblastoma hypoxia signature (NB-hypo) [29, 39] and we demonstrated that NB-hypo is an independent risk factor for neuroblastoma patients [12]. The importance of hypoxia and hypoxia inducible genes in the progression, differentiation and spreading of neuroblastoma has been the subject of several reports [12, 34, 40–42].

Here, we describe a robust classifier, based on NB-hypo, predicting neuroblastoma patients’ outcome with a very low error rate.

## Methods

### Patients

**Fig. 1:**
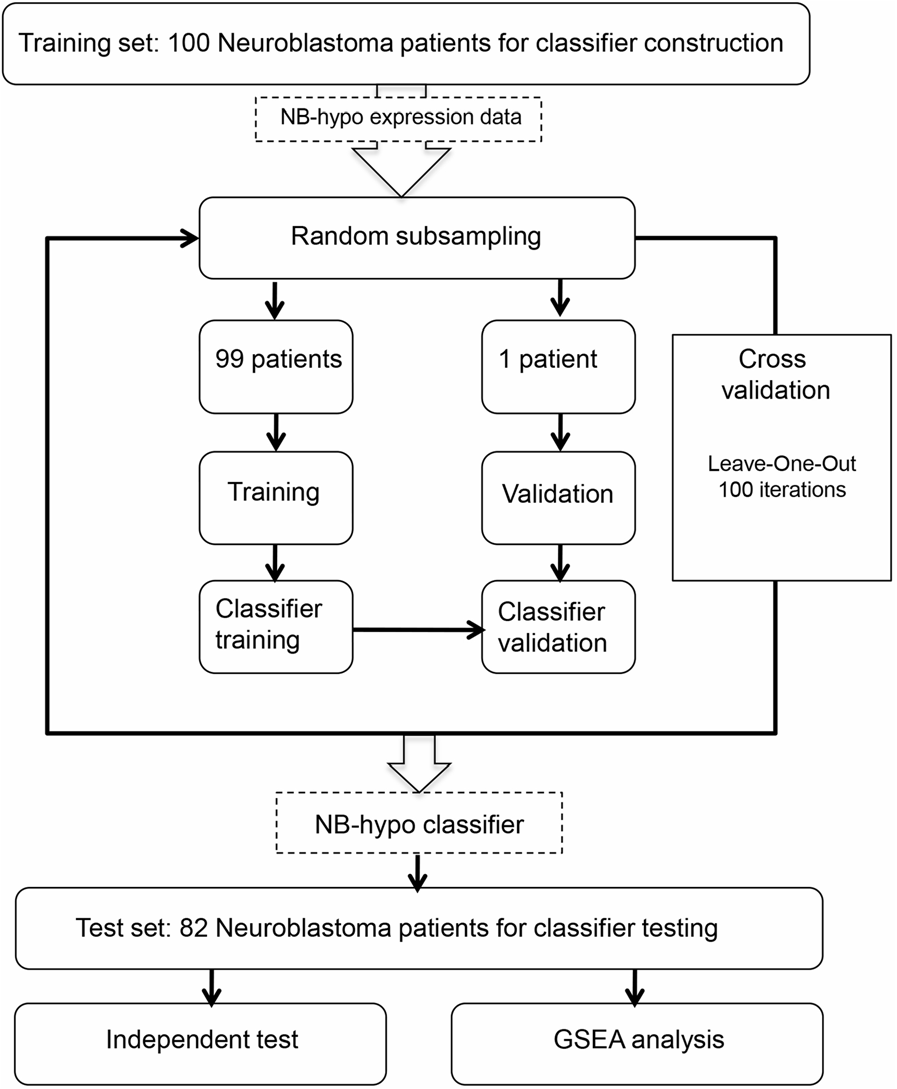
Schematic representation of the procedures used to build the NB-hypo classifier. The gene expression of 182 neuroblastoma tumors was measured by microarray on Affymetrix GeneChip HG-U133plus2.0. The dataset was divided into training (100 patients) and test (82 patients) sets. ANN model was applied to the training set in a 100 loops cross-validation scheme. The classifier was then applied to the test set. GSEA evaluated the enrichment of hypoxia related gene sets in the groups defined by the NB-hypo classifier

A total of 182 neuroblastoma patients belonging to four independent cohorts were enrolled on the basis of the availability of gene expression profile by Affymetrix GeneChip HG-U133plus2.0 and clinical and molecular information. Eighty-eight patients were collected by the Academic Medical Center (AMC; Amsterdam, Netherlands) [12, 43]; 21 patients were collected by the University Children’s Hospital, Essen, Germany and were treated according to the German Neuroblastoma trials, either NB 97 or NB 2004; 51 patients were collected at Hiroshima University Hospital or affiliated hospitals and were treated according to the Japanese neuroblastoma protocols [44]; 22 patients were collected at Gaslini Institute and were treated according to Associazione Italiana Ematologia e Oncologia Pediatrica (AIEOP) or International Society of Pediatric Oncology Europe Neuroblastoma (SIOPEN) protocols. The data are stored in the R2 repository (http://r2.amc.nl) or in the BIT-NB Biobank of the Gaslini Institute. Informed consent was obtained in accordance with institutional policies in use in each country. Tumor samples were obtained before treatment at the time of diagnosis. Median follow-up was longer than 5 years. Tumor stage was defined according to the International Neuroblastoma Staging System [45]. We randomly divided the cohort in two groups of 100 and 82 patients. We utilized the expression data of 100 tumors in a leave-one-out analysis to select and construct the classifier and the expression data of the remaining 82 tumors constituted the external test dataset (Fig. 1). The clinical characteristics of the 182 neuroblastoma tumors are detailed in Table 1. Good and poor outcome were defined as patient’s status (alive or dead) 5 years after diagnosis.

**Table 1:** Neuroblastoma patient’s dataset

Patients’ characteristics	Training set^a^	Test set^a^
Age at diagnosis^b^		
< 1 year	50 (50 %)	36 (44 %)
≥ 1 year	50 (50 %)	46 (56 %)
INSS stage^c^		
1,2,3,4s	67 (67 %)	49 (60 %)
4	33 (33 %)	33 (40 %)
MYCN status^d^		
normal	84 (84 %)	68 (83 %)
amplified	16 (16 %)	14 (17 %)
Outcome^e^		
Good	72 (72 %)	59 (72 %)
Poor	28 (28 %)	23 (28 %)

^a^The 182 patients’ dataset is split into two groups of 100 and 82 patients representing the training and test set, respectively

The total number of patients and the relative percentage in each subdivision is shown

^b^Age at diagnosis is defined as the patient’s age before or after 1 year

^c^INSS stage is defined according to the International Neuroblasma Staging System (INSS) [2]

INSS divided tumors into 5 stages (1,2,3,4,4s)

Stage 1 indicates localised tumour with incomplete gross excision; representative ipsilateral non-adherent lymph nodes negative for tumour microscopically. Stage 2 indicates localised tumour with or without complete gross excision, with ipsilateral non-adherent lymph nodes positive for tumour. Enlarged contralateral lymph nodes should be negative microscopically. Stage 3 indicates unresectable unilateral tumour infiltrating across the midline, with or without regional lymph node involvement; or localised unilateral tumour with contralateral regional lymph node involvement; or midline tumour with bilateral extension by infiltration (unresectable) or by lymph node involvement. Stage 4 indicates any primary tumour with dissemination to distant lymph nodes, bone, bone marrow, liver, skin, or other organs (except as defined by stage 4s). Stage 4s indicates localised primary tumour in infants younger than 1 year with dissemination limited to skin, liver, or bone marrow

^d^The status of the N-myc proto-oncogene is defined as amplified or normal according to the copy number of the gene on chromosome 2

^e^Good and poor outcome were defined as patient’s status (alive or dead) 5 years after diagnosis

### Gene expression analysis

Gene expression profiles for the 182 tumors were obtained by microarray experiment using Affymetrix GeneChip HG-U133plus2.0 [46] and the data were processed by MAS5.0 software according Affymetrix’ s guideline.

### Classifiers

Multi-Layer Perceptron (MLP) is a feedforward artificial neural network (ANN). MLP was trained on the expression values of the 62 probe sets constituting NB-hypo signature [12] to develop a predictive model for neuroblastoma patients’ outcome.

ANNs are organized in a number of input nodes, representing the attributes in the data, one or more hidden layers, where each layer is composed by a number of processing elements (hidden units), and one or more output nodes representing the output of the network. The input nodes receive the input data as a vector of variables and this information is passed through to the units in the first hidden layer and processed by a set of associated weights. Each hidden node calculates the output as follows [47]:

$$ {\displaystyle {v}_k}={\displaystyle \sum_{i=1}^n{\displaystyle {w}_{k_i}}}{\displaystyle {x}_i} $$

and

$$ {\displaystyle {y}_k}=\Phi \left({\displaystyle {v}_k}+{\displaystyle {v}_{{}_{{\displaystyle {k}_0}}}}\right) $$

where *x*_1,…,_ *x*_*n*_ are input variables, converging to the unit *k. w*_*k*__1,…,_ *w*_*k*__*n*_ are the weights connecting unit *k. v*_*k*_ is the net input. *y*_*k*_ is the output of the unit where *v*_*k*__0_ is a bias term and Φ(⋅) is the activation function commonly of the form:

$$ \Phi (v)=\frac{1}{1+{\displaystyle {e}^{-v}}} $$

for the sigmoid activation function. Ultimately, the modified information reaches the output nodes as output of the ANN.

ANNs are trained to be capable of accurately modeling a set of examples and predicting their output [47]. The backpropagation training algorithm is a computationally straightforward algorithm for training the multi-layer perceptron [48], which uses the gradient descent procedure to find the combination of weights, resulting in the smallest error [48]. A learning rate controls the size of the weights changes and a momentum term prevents the network in becoming trapped in local minima, or being stuck along flat regions in error space [47]. Regularization techniques are applied to prevent the risk of low generalization ability [47]. One commonly used regularization technique stops the training process when a predetermined number of iterations have completed.

We set up a three-layer neural network architecture containing a single hidden layer with 32 hidden units. The number of hidden units is calculated as the fraction between, the sum of the number of probe sets and the number of outcomes, and two. The activation function of the hidden layer units was the sigmoid function. We scaled data for improving the performance of the network. We utilized the back-propagation process with learning rate and momentum set to 0.3 and 0.2, respectively. The predetermined maximum number of iterations was set to 500.

The Support Vector Machine (SVM) [49], the Logistic regression (LOR) [50], and the Naïve Bayesian (NAB) [51] algorithms were also utilized for classification. LibSVM implementation of SVM was ran with homogeneous polynomial kernel, degree of the polynomials set to 3, gamma parameter set to 0.05 and tolerance of the termination criterion set to 0.001.We ran NAB with no supervised discretization and no kernel estimator for numeric attributes and LOR with ridge parameter set to 1.0e-7 and Broyden–Fletcher–Goldfarb–Shanno (BFGS) regularization.

The algorithms were implemented by the Waikato Environment for Knowledge Analysis (WEKA) software version 3.7.10 [52].

### Metrics

Let *TP* to be the number of true positives, *TN* the number of true negatives, *FP* the number of false positives and *FN* the number of false negatives in a confusion matrix, we defined good outcome as positive and poor outcome as the negative.

Accuracy, sensitivity, precision, specificity, negative predictive value (NPV), Matthew’s Correlation Coefficient (MCC) and F1-score metrics measured the performance of the classifier.

Accuracy measures the proportion of correctly classified patients [53] and it is calculated by the formula:

$$ Accuracy=\frac{TP+TN}{TP+FP+TN+FN} $$

Sensitivity, also named True Positive Rate or Recall, measures the proportion of good outcome patients correctly classified as such [53] and it is calculated by the formula:

$$ Sensitivity=\frac{TP}{TP+FN} $$

Precision measures the proportion of correctly classified good outcome patients [53] and it is calculated by the formula:

$$ Precision=\frac{TP}{TP+FP} $$

Specificity measures the proportion of poor outcome patients correctly classified as such [53] and it is calculated by the formula:

$$ Specificity=\frac{TN}{TN+FP} $$

NPV measures the proportion of correctly classified poor outcome patients. NPV is calculated by the formula:

$$ NPV=\frac{TN}{TN+FN} $$

MCC measures the correlation between a classifier prediction and the observed outcomes. We calculated MCC by the formula:

$$ MCC=\frac{\left(TP*TN\right)-\left(FP*FN\right)}{\sqrt{\left(TP+FP\right)\left(TP+FN\right)\left(TN+FP\right)\left(TN+FN\right)}} $$

When MCC equals 0, the performance is comparable with that of a random prediction.

F1-score measures the weighted average of the precision and sensitivity. We calculated the F1-score by the formula:

$$ F1-score=2\frac{Precision\ast Sensitivity}{Precision+ Sensitivity} $$

### Statistical analysis

We estimated the probability of overall survival (OS) and event-free survival (EFS) using the Kaplan-Meier method, and we measured the significance of the difference between Kaplan-Meier curves by log-rank test using Prism 6.1 (GraphPad Software, Inc.). Independence among the clinical variables and NB-hypo prediction was assessed by multivariate cox analysis. MYCN status, INSS stage and Age at diagnosis were included in the analysis as binary variables.

### Gene set enrichment analysis

We utilized the GSEA [54] to evaluate the enrichment of hypoxia related gene sets in patients predicted with “Poor” or “Good” outcome. We carried out the analysis on all probe sets of the HG-U133 Plus 2.0 GeneChip. GSEA calculates an enrichment score (ES) and normalized enrichment score (NES) for each gene set and estimates the statistical significance of the NES by an empirical permutation test using 1.000 gene permutations to obtain the nominal *p*-value. However, when multiple gene sets are evaluated, GSEA adjusts the estimate of the significance level to account for multiple hypothesis testing. To this end, GSEA computes the False Discovery Rate q-value (FDR q-value) measuring the estimated probability that the normalized enrichment score represents a false positive finding [54]. The gene sets used in the analysis belong to the Chemical and genetic perturbation (C2.CGP) collection of the Molecular Signature Database (MSigDB) v5 database [54]. We selected 14 gene sets related to the hypoxia response from the C2.CGP collection using “hypoxia” as keyword and containing between 20 and 300 probe sets (see Additional file 1). FDR q-value smaller than 0.25 is considered significant.

## Results

We analyzed the gene expression of 182 neuroblastoma tumors profiled by the Affymetrix HG-U133plus2.0 platform [46]. The clinical characteristics of the 182 neuroblastoma patients are detailed in the Table 1. “Good” or “poor” outcome is defined, from here on, as the patient’s status “alive” or “dead” 5 years after diagnosis, respectively. We randomly divided the cohort into two groups of 100 (55 %) and 82 (45 %) patients to create the training and test set, respectively (Fig. 1). We utilized the expression data of the training set to construct the classifier and the leave-one-out approach to measure the performance of the algorithms. The classifier was then tested on the independent 82 patients dataset. We previously described a 62 probe sets signature that represents the hypoxic response of neuroblastoma cell lines [29] (NB-hypo) and we used this signature to develop a hypoxia-based classifier to predict the patients’ outcome (NB-hypo classifier).

To this end, we compared the performances of Multi-layer perceptron (MLP), Support Vector Machine (SVM), Logistic regression (LOR), and Naïve Bayesian (NAB) algorithms in classifying neuroblastoma patients’ outcome. We evaluated the classification by measuring accuracy, sensitivity, precision, specificity, negative predictive value, Matthew’s correlation coefficient and F1-score indicators by leave-one-out cross validation. The results (see Additional file 2: Table S1) showed that MLP performed similarly or better than the other algorithms tested depending on the indicator and MLP was chosen to generate the NB-hypo classifier.

We tested the MLP classifier on an independent test set of 82 neuroblastoma patients and we found that it predicted correctly 53/59 (90 %) good outcome and 18/23 (78 %) poor outcome patients, resulting in an accuracy of 87 % (Fig. 1).

We compared the performance of NB-hypo classifier with that of the known neuroblastoma risk factors: age at diagnosis, INSS stage and MYCN status by subdividing the patients of the test set according to these risk factors and calculating the prediction performances (Table 2). NB-hypo classifier achieved the highest predictive accuracy (87 %) and MCC (67 %) compared to the other risk factors (ranging from 72 to 84 % for accuracy and from 48 to 58 % for MCC). MYCN status had the highest sensitivity and NPV, but the lowest specificity and precision whereas age at diagnosis showed the opposite trend indicating strong phenotype biases of these risk factors. In contrast, NB-hypo classifier and INSS stage obtained a more balanced specificity and sensitivity indicating a less biased classification error distribution between good and poor outcome. NB-hypo classifier and MYCN had the highest F1-score indicating the good balance of sensitivity and precision of these two factors.

**Table 2:** NB patients classification by different risk factors

	Performance^a^						
Predictor	Accuracy^b^	Sensitivity^c^	Precision^d^	Specificity^e^	NPV^f^	MCC^g^	F1-score^h^
NB-hypo classifier (Good vs Poor)	87 %	90 %	91 %	78 %	75 %	67 %	90 %
Age at diagnosis (< 1 year vs ≥ 1 year)	72 %	61 %	100 %	100 %	50 %	55 %	76 %
INSS stage (1,2,3,4s vs 4)	76 %	75 %	90 %	78 %	55 %	78 %	82 %
MYCN status (normal vs amplified)	84 %	97 %	84 %	52 %	86 %	58 %	90 %

^a^Performance of NB-hypo classifier and other commonly used neuroblastoma risk factors in the test set

For prediction of prognosis by age at diagnosis, patients older than one year were predicted with poor prognosis. For prediction by stage, patients with stage 1,2,3, and 4s were predicted with good prognosis and patients with stage 4 were predicted with poor prognosis. For prediction by MYCN status, patients with amplified MYCN were predicted with poor prognosis while patients without MYCN amplification were predicted with good prognosis

^b^Accuracy measures the proportion of correctly classified patients

^c^Sensitivity measures the proportion of good outcome patients correctly classified as such

^d^Precision measures the proportion of correctly classified good outcome patients

^e^Specificity measures the proportion of poor outcome patients correctly classified as such

^f^NPV(Negative Predictive Value) measures the proportion of correctly classified poor outcome patients

^g^MCC (Matthew's correlation coefficient) measures the correlation between a classifier prediction and the observed outcomes

^h^F1-score measures the weighted average of the precision and sensitivity

**Fig. 2:**
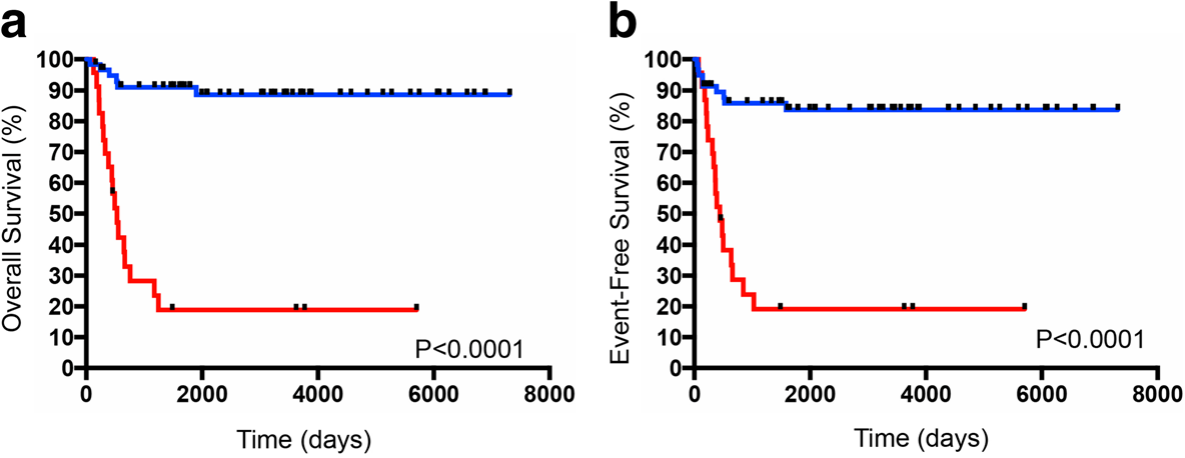
Kaplan-Meier and log-rank analysis for the 82 neuroblastoma patients belonging to the external test dataset. Overall survival (**a**) and event free survival (**b**) of patients classified according to the NB-hypo classifier. Red and blue curves represent predicted Poor and Good outcome patients, respectively. The *p*-value of the log-rank test is shown

The overall and event free survival of the patients divided according to the NB-hypo classifier are shown in Fig. 2. Kaplan-Meier curves and log-rank test demonstrated that patients with Good and Poor outcome prediction had a significantly different survival (*p* < 0.0001). In addition, NB-hypo classifier is an independent predictor of overall survival and event free survival (*p* < 0.05) of neuroblastoma patients when compared to the common risk factors INSS stage, Age at diagnosis, and MYCN status in a multivariate cox analysis (Table 3). We concluded that NB-hypo classifier was an independent prognostic factor for neuroblastoma and very accurate in predicting the outcome of neuroblastoma patients relative to other prognostic markers.

**Table 3:** Multivariate Cox analysis results of the test set

	Multivariate cox analysis (OS)^a^				Multivariate cox analysis (EFS)^b^			
Covariate	Coefficient^c^	HR^d^	95 % Cl^e^	*P*-value^f^	Coefficient^c^	HR^d^	95 % Cl^e^	*P*-value^f^
NB-hypo classifier (Good vs Poor)	1.1	3.3	(1.0, 10.6)	4.00E-02	1.1	3	(1.0, 9.0)	4.00E-02
Age group (<12 months vs ≥ 12 months)	1.9	4E-08	(0.0, inf)	9.90E-01	1.2	3.6	(0.9, 14.2)	6.00E-02
INSS stage (1,2,3,4s vs 4)	0.6	1.9	(0.5, 6.4)	2.70E-01	0.4	1.5	(0.5, 4.5)	4.00E-04
MYCN status (nomal vs amplified)	0.3	1.3	(0.5, 3.5)	4.90E-01	0.4	1.5	(0.6, 3.9)	3.00E-01

^a^Multivariate cox regression analysis for overall survival

^b^Multivariate cox regression analysis for event - free survival

^c^Cox regression coefficient

^d^Hazard ratio

^e^95 % of confidence interval

^f^Significance. Values smaller than 0.05 are acceptable

**Fig. 3:**
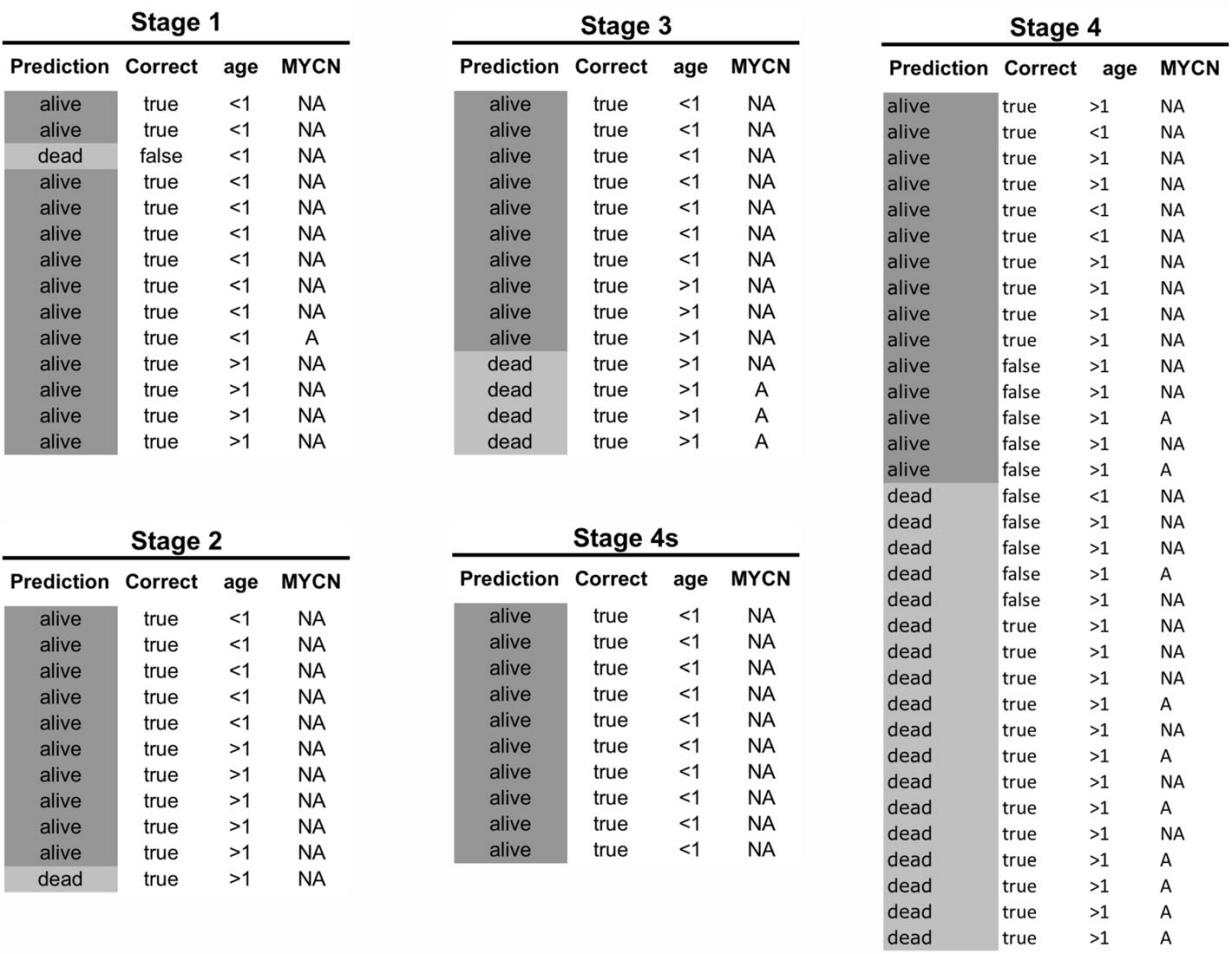
The plot shows the concordance between NB-hypo prediction and the clinical characteristics of the 82 patients in the external test dataset. Patients are grouped according to INSS staging. Rows represent individual patients. For each stage, the column “Prediction” indicates the prediction results of NB-hypo classifier (Poor or Good). The column “Correct” represents the correctness of NB-hypo classifier prediction (true or false). The column “Age” shows the age at diagnosis (>1 year vs. < 1 year). The column “MYCN” shows the MYCN amplification status (A = amplified; NA = not amplified). Patients marked with a clearer color are the ones predicted as “Poor” by NB-hypo classifier

We assessed the concordance between NB-hypo prediction and patients’ characteristics (Fig. 3). We divided the patients by INSS stage reporting for each group the outcome prediction by NB-hypo classifier, the concordance between the prediction and the outcome, age at diagnosis and MYCN status. Interestingly, we found the good 98 % concordance (48/49) between patient’s outcome and prediction in localized (stage 1,2,3) and stage 4s tumors indicating that NB-hypo has 2 % classification error in non-stage 4 patients. This result is particularly interesting because the prediction was accurate in assessing the uncommon death of 5 low or intermediated risk patients. Among the correctly predicted patients, age at diagnosis and MYCN amplification status were evenly distributed (Fig. 3), demonstrating the independence between these risk factors and the NB-hypo classifier and in agreement with results shown in Table 3. In contrast, the majority of misclassified patients belonged to stage 4, in agreement with the fact that prognosis of this stage is traditionally difficult [55]. Taken together, these results demonstrate that NB-hypo classifier is a powerful tool to predict neuroblastoma patients’ outcome.

We analyzed the hypoxic status of the tumors utilizing the gene set enrichment analysis (GSEA) [54]. We utilized GSEA to determine whether known sets of hypoxia-inducible genes were significantly enriched in the tumor gene expression profile in relationship to the “Poor” or “Good” outcome prediction. We studied 14 gene sets characteristic of the hypoxia response according to the literature and included in the GSEA MSigDB database (see Additional file 1 and Methods section for details). These gene sets were independently derived by other groups to assess the hypoxic status of various tissues different from neuroblastoma. Eleven hypoxia gene sets were significantly enriched in the patients classified as “dead” (FDR q-value < 0.25), whereas none was enriched in those classified as “alive”, demonstrating association between the poor outcome and the hypoxic status of the tumor (Table 4). We concluded that poor prognosis patients have a hypoxic phenotype.

**Table 4:** Hypoxia-related gene sets enriched in patients classified as Poor outcome

Gene set^a^	ES^b^	NES^c^	FDR q-value^d^
WINTER_HYPOXIA_UP	0.72	2.22	0.00
HARRIS_HYPOXIA	0.52	1.90	0.02
JIANG_HYPOXIA_CANCER	0.42	1.83	0.03
ELVIDGE_HYPOXIA_BY_DMOG_DN	0.46	1.76	0.03
NB-HYPO_62-PBSETS	0.53	1.65	0.06
WACKER_HYPOXIA_TARGETS_OF_VHL	0.60	1.61	0.06
KRIEG_HYPOXIA_VIA_KDM3A	0.42	1.64	0.06
KIM_HYPOXIA	0.48	1.59	0.06
MENSE_HYPOXIA_UP	0.44	1.58	0.05
LEONARD_HYPOXIA	0.45	1.47	0.08
WEINMANN_ADAPTATION_TO_HYPOXIA_DN	0.36	1.19	0.24

^a^Hypoxia-related gene sets enriched in the GSEA analysis

^b^ES (enrichment score) is the maximum deviation from zero encountered in a random walk for a gene set

^c^NES (normalized enrichment score) is the fraction between the ES and the mean of the ES against a number of permutations of the dataset

^d^FDR q-value is the estimated probability that the normalized enrichment score represents a false positive finding. Values <= 0.25 are considered acceptable

## Discussion

We developed a classifier based on tumor gene expression that predicts neuroblastoma patients’ outcome with high accuracy. We utilized a bottom up, biology-driven, approach [12], which is based on the prior knowledge of the influence of tumor hypoxia on neuroblastoma growth. One advantage of this strategy is the immediate appreciation of the molecular program related to the prognostic indication [12, 56]. This process followed a rigorous sequence starting from the definition of neuroblastoma hypoxic response signature in tumor cell lines [29] followed by the demonstration that this signature is an independent risk factor [12] and the findings, reported here, that the MLP, applied to the 62 probe sets of the signature generates a robust outcome and tumor hypoxia predictor with potential clinical applications.

The importance of hypoxia in conditioning tumor aggressiveness is documented by an extensive literature [30, 32–34, 36, 37, 57]. Studies on the relationship between hypoxia inducible factors and neuroblastoma aggressiveness showed that high HIF-2 alpha expression correlated with disseminated disease (for review see [58]). However, there is little information on the potential of hypoxia as a biomarker for patients’ stratification possibly because it is difficult of quantifying hypoxia, patchy in nature, in a tumor mass [59]. Microarray technology, applied to tumors, has the potential to overcome this difficulty and to provide a probe to monitor average hypoxia in the tumor mass [60]. The use of gene expression signatures to measure hypoxia has been reported [36, 56, 61] and their potential as prognostic factors was shown, for example, in soft tissues sarcomas [62] and hepatocellular carcinoma [63].

Several statistical and machine learning techniques can be used for classification [64, 65]. Here, we described the successful application of the multi-layer perceptron for NB patients’ outcome prediction. MLPs are a form of machine learning with proven pattern recognition capabilities that were utilized in many areas of bioinformatics such as disease classification and identification of biomarkers [47]. MLP demonstrated a similar/better performance relative to SVM, NAB and LOR algorithms proving to be a robust tool for the analysis of complex gene expression data.

Utilizing the MLP algorithm with the NB-hypo signature previously described [12], we generated a robust and independent classifier capable of stratifying patients with distinct overall and event-free survival and predicting patients’ good or poor outcome with 87 % accuracy of and 67 % MCC. These values are better than what can be achieved with other available risk factors (MYCN amplification, age at diagnosis and INSS stage) on the same cohort. These findings extend and complement previous work on NB patients’ classifiers based on Logic Learning machine (LLM) [11, 66] trained through an optimized version of the Shadow Clustering algorithm [67]. These studies were instrumental to demonstrate that hypoxia based predictors could generate intelligible rules translatable into the clinical settings [66]. However, the feature selection system of LLM reshaped the feature space definition for optimizing the rule construction and only a fraction of the NB-hypo probe sets was tested in these studies. The present work provides novel and critical evidence that the 62 probe sets of the NB-hypo signature will work as a whole, providing robustness to the classifier generated by application of the Multi-layer Perceptron algorithm.

Several groups have used gene expression-based approaches to stratify neuroblastoma patients and prognostic gene signatures have been described [11, 13–22]. The performance of our NB-hypo classifier is comparable with that of the other prognostic gene expression signatures proposed for neuroblastoma [68]. However, some of them were obtained by supervised computational methods applied to the entire gene expression profile of primary tumors or by meta-analysis of existing data. These approaches generated interesting results but the signatures, and the resulting classifiers, have some limitations. On one hand, these gene signatures have little overlap because of the high variability of the tumor data sets. On the other, it is difficult to interpret the results with respect to the underlying biology because the assembly of the signature is purely mathematical. Finding a predictor that can be linked to molecular mechanisms of cancer development is critical for translating these markers to the clinic. One added value of our predictor is that the choice of a biology driven approach links our tumor selection to the hypoxia molecular program that can be associated to the progress of the disease and exploited to manage the neuroblastoma.

When we evaluated the concordance between NB-hypo prediction and INSS stage, we found that NB-hypo correctly predicted the status of almost all patients with localized or 4s stage tumors. More importantly, we identified, in this group, all patients with poor outcome that may benefit from a more aggressive, and perhaps hypoxia related treatment. Validation of this conclusion on additional data sets is required.

The suggestion of developing hypoxia-related treatments relies on the demonstration that poor outcome tumors are hypoxic. The expression of the NB-hypo signature is the first line of evidence in this respect. The GSEA analysis was an independent strategy to explore the relationship between NB-hypo outcome prediction and tumor hypoxia because it is based on the analysis of all forty thousand probe sets of the tumor expression profile. GSEA measures the representation of hypoxia-related gene sets coming from independent, published studies in the good or poor prognosis patients. We demonstrated a great and selective enrichment of hypoxia related gene sets in a large group of poor outcome patients.

The characterization of the tumor at diagnosis is indispensable for deciding the treatment and the NB-hypo classifier poor outcome prediction may identify the tumors that, as a result of the hypoxic status, express high genetic instability [69], contain undifferentiated or cancer stem cells [32, 40] or a higher metastatic potential [33, 34]. Therapeutic agents are being developed to target hypoxia (for review see [59]) and are being tested in the clinic. Our classifier may be instrumental for their application to neuroblastoma.

## Conclusions

We developed a robust classifier predicting neuroblastoma patient’s outcome with a very low error rate and we provided independent evidence that the poor outcome tumors are hypoxic, supporting the potential of using hypoxia as target for neuroblastoma treatment. The definitive validation of hypoxia as a prognostic factor in clinical trials rests on the possibility to analyze a larger dataset to validate the existence of small group of patients, with unique clinical history, in which tumor hypoxia may be the driving force to poor outcome. We will look at the potential of cross platform approaches to compare and utilize existing neuroblastoma gene expression dataset obtained with different platforms. This task is not easy but it is feasible and promises to assemble a significant number of cases for improving the predictive value of hypoxia-related signatures in neuroblastoma.

A second way to boost the robustness of the prediction is to increase the spectrum of molecular data associated to the patient. Ribonucleic acid (RNA) assessment by microarray analysis is becoming an affordable and reliable method to characterize hypoxia response. However, microRNAs, non coding RNA, protein patterns, transcription factors analysis, promise to generate equally important information to define the biology of tumor hypoxia. The full exploitation of this wealth of data will require a parallel bioinformatics effort to develop the relevant multiplatform pathway analysis and studies along this way are in progress.

## Additional files

Additional file 1: Gene sets utilized in the GSEA analysis. The table shows a list of 14 hypoxia-related gene sets and the relative number of probe sets. The gene sets used in the analysis belong to the C2.CGP collection and were obtained by the GSEA MSigDB v5 database [54]. The gene sets were selected inputting the keyword “Hypoxia” in the MSigDB and filtering out those having less than 20 probe sets and more 300 probe sets. (PDF 58 kb)
Additional file 2: Performance of learning algorithms in neuroblastoma patients’ classification. The table shows the performance of MLP (multi-layer perceptron), SVM (support vector machine), LOR (logistic regression) and NAB (naïve Bayesian) algorithms assessed by leave-one-out cross validation in the training set. (PDF 60 kb)

## Abbreviations

AIEOP: Associazione Italiana Ematologia e Oncologia Pediatrica
AMC: Academic Medical Center
ANN: Artificial Neural Networks
CGP: Chemical and genetic perturbation
DNA: Deoxyribonucleic acid
EFS: Event-free survival
ES: Enrichment Score
FDR: False discovery rate
GSEA: Gene set enrichment analysis
HIF: Hypoxia inducible factor
INSS: International neuroblastoma staging system
LLM: Logic learning machine
LOR: Logistic regression
MCC: Matthew’s correlation coefficient
MLP: Multi-layers perceptron
MSIGDB: Molecular Signature Database
MYCN: Myelocytomatosis viral related oncogene Neuroblastoma derived
NAB: Naïve Bayesian
NB: Neuroblastoma
NES: Normalized enrichment score
NPV: Negative predictive value
OS: Overall survival
RNA: Ribonucleic acid
SIOPEN: International society of pediatric oncology europe neuroblastoma
SVM: Support vector machine
WEKA: Waikato environment for knowledge analysis
