# Supplementary material for: Artificial neural network classifier predicts neuroblastoma patients’ outcome
Source: BMC Bioinformatics. 2016 Nov 8;17(Suppl 12):83–93. doi: 10.1186/s12859-016-1194-3 (PMC5123344; doi:10.1186/s12859-016-1194-3)
Supplement: Additional file 1: — Gene sets utilized in the GSEA analysis. The table shows a list of 14 hypoxia-related gene sets and the relative number of probe sets. The gene sets used in the analysis belong to the C2.CGP collection and were obtained by the GSEA MSigDB v5 database [54]. The gene sets were selected inputting the keyword “Hypoxia” in the MSigDB and filtering out those having less than 20 probe sets and more 300 probe sets. (PDF 58 kb) [file 12859_2016_1194_MOESM1_ESM.pdf]

Additional file 1

Table S1 – Gene sets utilized in the GSEA analysis

Gene set[a]	No of probe sets[b]
NB-HYPO_HYPOXIA	62
WINTER_HYPOXIA_UP	127
WINTER_HYPOXIA_DN	67
ELVIDGE_HYPOXIA_BY_DMOG_DN	140
MENSE_HYPOXIA_UP	129
KIM_HYPOXIA	57
HARRIS_HYPOXIA	217
LEONARD_HYPOXIA	106
JIANG_HYPOXIA_CANCER	196
JIANG_HYPOXIA_VIA_VHL	99
WACKER_HYPOXIA_TARGETS_OF_VHL	24
KRIEG_HYPOXIA_VIA_KDM3A	142
WEINMANN_ADAPTATION_TO_HYPOXIA_UP	74
WEINMANN_ADAPTATION_TO_HYPOXIA_DN	102

[a] Gene sets utilized in the GSEA analysis

[b] Number of probe sets in the gene set
