# Supplementary material for: Artificial neural network classifier predicts neuroblastoma patients’ outcome
Source: BMC Bioinformatics. 2016 Nov 8;17(Suppl 12):83–93. doi: 10.1186/s12859-016-1194-3 (PMC5123344; doi:10.1186/s12859-016-1194-3)
Supplement: Additional file 2: — Performance of learning algorithms in neuroblastoma patients’ classification. The table shows the performance of MLP (multi-layer perceptron), SVM (support vector machine), LOR (logistic regression) and NAB (naïve Bayesian) algorithms assessed by leave-one-out cross validation in the training set. (PDF 60 kb) [file 12859_2016_1194_MOESM2_ESM.pdf]

Additional file 2

Table S1 - Performance of different classification algorithms

	Performance[a]						
Predictor	Accuracy[b]	Sensitivity[c]	Precision[d]	Specificity[e]	NPV[f]	MCC[g]	F1-score[h]
MLP	84%	90%	88%	68%	73%	60%	89%
SVM	80%	86%	86%	64%	64%	50%	86%
LOR	78%	82%	87%	68%	59%	48%	84%
NAB	75%	87%	80%	43%	57%	34%	83%

[a] Performance of MLP (multi-layer perceptron), SVM (support vector machine), LOR (logistic regression), NAB (naïve bayesian) algorithms assessed by leave-one-out cross-validation in the training set.

[b] Accuracy measures the proportion of correctly classified patients.

[c] Sensitivity measures measures the proportion of good outcome patients correctly classified as such.

[d] Precision measures the proportion of correctly classified good outcome patients.

[e] Specificity measures the proportion of poor outcome patients correctly classified as such..

[f] NPV(Negative Predictive Value) measures the proportion of correctly classified poor outcome patients.

[g] MCC (Matthew's correlation coefficient) measures the correlation between a classifier prediction and the observed outcomes.

[h] F1-score measures the weighted average of the precision and sensitivity.
